# Influence of an iron dextran injection in various diseases on hematological blood parameters, including serum ferritin, neonatal dairy calves

**DOI:** 10.1186/s12917-024-04229-y

**Published:** 2024-08-24

**Authors:** Marlene Sickinger, Jessica Joerling, Kathrin Büttner, Joachim Roth, Axel Wehrend

**Affiliations:** 1 Clinic for Ruminants and Herd Health Management, Justus-Liebig-University of Giessen, Frankfurter Str. 104, 35392 Giessen, Germany; 2 Veterinary Practice Lünne, Lünne, Germany; 3 Department for Biomathematics and Data Processing, Justus-Liebig-University of Giessen, Giessen, Germany; 4 Institute for Veterinary Physiology, Justus-Liebig-University of Giessen, Giessen, Germany; 5 Veterinary Clinic for Reproduction and Neonatology, Justus-Liebig-University of Giessen, Giessen, Germany

**Keywords:** Iron homeostasis, Cattle, Anemia, Serum ferritin, Serum iron

## Abstract

**Background:**

Feeding milk substitutes with low iron content or whole milk without iron supplementation is considered a major factor in developing iron-deficiency anemia in neonatal dairy calves. Young calves are often supplemented with iron dextran injections on the first day of life to prevent anemia. However, the effects of preventive treatment and the presence of disease on serum iron (Fe) concentrations, serum ferritin levels, and hematological blood parameters during the early neonatal stages have not been examined in detail. Therefore, we examined and evaluated the effects of iron dextran injections and health status on the development of hematocrit (Ht), red blood cells (RBC), hemoglobin concentration (Hb), erythrocyte indices (mean corpuscular volume, mean corpuscular hemoglobin, mean corpuscular hemoglobin concentration), Fe, and serum ferritin concentrations in dairy calves within the first 10 days of life. The suitability of serum ferritin as a reliable indicator of anemia in very young calves was evaluated by correlating ferritin concentrations with known laboratory diagnostic parameters of anemia.

**Results:**

Iron supplementation significantly increased Fe levels (*P* = 0.048) but did not affect serum ferritin levels in neonatal calves. Fe concentrations were significantly lower in diseased than healthy calves (*P* = 0.0417). Iron supplementation significantly affected the health status, as observed in Ht (P_treat_=0.0057; P_health_=0.0097), RBC (P_treat_=0.0342; P_health_=0.0243), and Hb (P_treat_=0.0170; P_health_=0.0168). Serum ferritin levels did not significantly correlate with Fe levels. Both groups showed marked differences in ferritin levels, with the highest levels measured on day 2. Fe concentrations showed weak negative correlations with Hb and Ht levels on day 3 (ρ=-0.45; *P* = 0.0034 and ρ=-0.045; *P* = 0.0032, respectively). RBC count showed strong positive correlations with Hb and Ht levels (ρ = 0.91 and ρ = 0.93; *P* < 0.001).

**Conclusion:**

Iron dextran injections increased Fe concentrations but reduced Ht level, RBC count, and Hb level. The presence of diseases led to a reduction in Fe and higher values of Ht, RBC, and Hb in moderate disease than in severe disease. Due to physiological fluctuations during the first 3 days of life, serum ferritin level seems unuseful for evaluating iron storage before day 4 of life.

## Background

The essential trace element, iron, is involved in several physiological processes, such as erythropoiesis, hemoglobin synthesis, oxygen transport, and enzyme functions [1–3]. Dairy calves fed whole milk or milk substitutes with low iron concentrations mainly exhibit iron deficiency and subsequent iron-deficiency anemia [1, 4].

Non-ruminating calves require an estimated 150 mg/kg dry matter (DM) of iron daily, whereas the requirement decreases to 24 mg/kg DM in ruminating cattle [2]. In adult cattle, iron deficiency is rare because roughage has a high iron content due to soil contamination [5, 6]. Iron-containing forage should be supplemented as soon as possible to reduce the risk of anemia in calves, or milk substitutes with iron supplements should be fed. However, milk substitutes containing 100 mg/kg DM of iron leads to declining iron levels in dairy calves over the first 7 weeks of life. Nevertheless, this decline does not negatively affect the hematological parameters [7]. Because of the uncertainty associated with oral supplementation, general prophylactic parenteral iron supplementation is generally recommended under field conditions.

However, an excessive supply of iron can negatively affect cellular metabolism through the formation of oxygen radicals [2, 8]. It has also been shown that disease alters iron homeostasis through a controlled reduction in iron concentration in the organism via internalization or reduction in resorption [9–15]. Therefore, the laboratory diagnosis of iron deficiency and anemia in newborn calves faces several challenges. Despite historical reports of anemia in calves, the diagnosis was mainly based on the concentrations of serum iron (Fe), hemoglobin (Hb), packed cell volume, and red blood cells (RBC) [16–18]. Recent studies on erythrocyte size ratios and other RBC parameters showed an increase in the number of erythrocytes in healthy calves, whereas their volume decreased by up to 20%. To avoid misinterpreting a decreasing mean corpuscular volume (MCV) in neonatal calves as a sign of iron-deficiency anemia, it is crucial to critically scrutinize the calf‘s age and the results of the clinical examination [7].

Evidence from human medicine indicates that the measurement of Hb alone is unsuitable for laboratory diagnostic confirmation of iron-deficiency anemia; therefore, ferritin (an iron storage protein) and transferrin are included as additional parameters in the diagnostic process [19]. Studies investigating ferritin levels in cattle are scarce and outdated [20–22]. Since the iron balance is strongly influenced by inflammation and infection, ferritin is also affected by the acute phase reaction. The release of cytokines leads to an increase in ferritin level [23] and can mask iron-deficiency anemia in the presence of inflammation [24].

The effects of preventive treatment and the presence of diseases (i.e. diarrhea) on Fe concentrations, serum ferritin levels, and hematological blood parameters during the early neonatal stages have not been examined in detail. Therefore, this study aimed to examine and evaluate the effects of iron dextran injections and health status on the development of hematocrit (Ht), RBC count, Hb concentration, erythrocyte indices (MCV, mean corpuscular hemoglobin [MCH], and mean corpuscular hemoglobin concentration [MCHC]), and Fe and serum ferritin concentrations in dairy calves within the first 10 days of life.

The suitability of serum ferritin as a reliable indicator of anemia in very young calves was also evaluated by correlating ferritin concentrations with known laboratory diagnostic parameters of anemia. We hypothesized that lower concentrations of standard anemia parameters would correlate with lower serum ferritin concentrations, thus resulting in a more sensitive diagnostic tool that might indicate deficiencies very early. The possible influence of sex on these parameters was also investigated in this study.

## Results

### Blood parameters

Common criteria for defining calves as anemic are reduced concentrations of Ht and Hb [1, 25]. Twenty-eight calves in our study displayed a reduction in concentrations of Ht (< 0.28 l/L) and Hb (< 5.6 mmol/L) during at least 1 day of the experimental period. Those 28 calves were 14 females and 14 males. RBC counts were reduced in only four calves for at least 1 day (< 5 T/L). Throughout the examination period, neither the MCV nor MCH values decreased in any of the calves. The MCHC was reduced (< 32.6 g/dL) in all 40 calves for at least 1 day during the first 10 days of life.

Reduced Fe concentrations were observed in 35 calves, and 24 of these calves were also deficient in Ht and Hb.

Ferritin concentrations decreased (< 15 ng/mL) in 33 calves. Additionally, 28 of these calves showed reduced Fe values and 19 showed reductions in Ht and Hb levels.

**Fig. 1:**
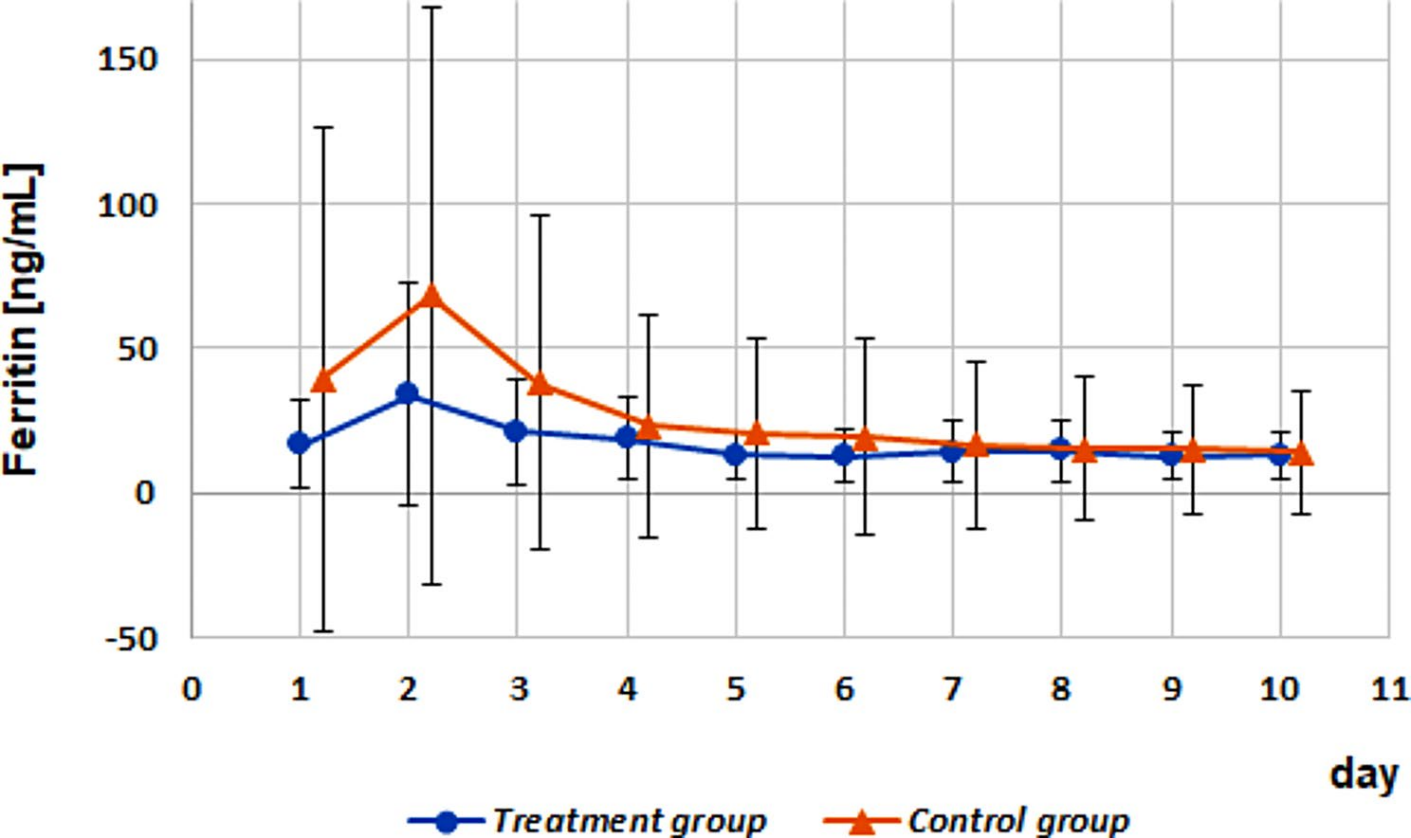
Timely course of x ± s for serum ferritin within the treatment group versus the control group. Dots and triangles mark the arithmetic means; whiskers present the standard deviations

In both groups, ferritin values peaked on day 2 and declined below the initial values until day 10. The ferritin concentrations in animals in the control group were remarkably higher than those in the treatment group (maxima: 68.17 ± 100.1 ng/mL vs. 34.2 ± 38.5 ng/mL; Fig. 1).

The ferritin area under the curve (AUC) values were not influenced by iron injection on day 1 (*P* = 0.9004), health status (*P* = 0.5032), or sex (*P* = 0.2714). There were no significant interactions among the fixed effects. No correlations were found between the AUC values of ferritin and those of Fe, RBC, Ht, or Hb.

**Fig. 2:**
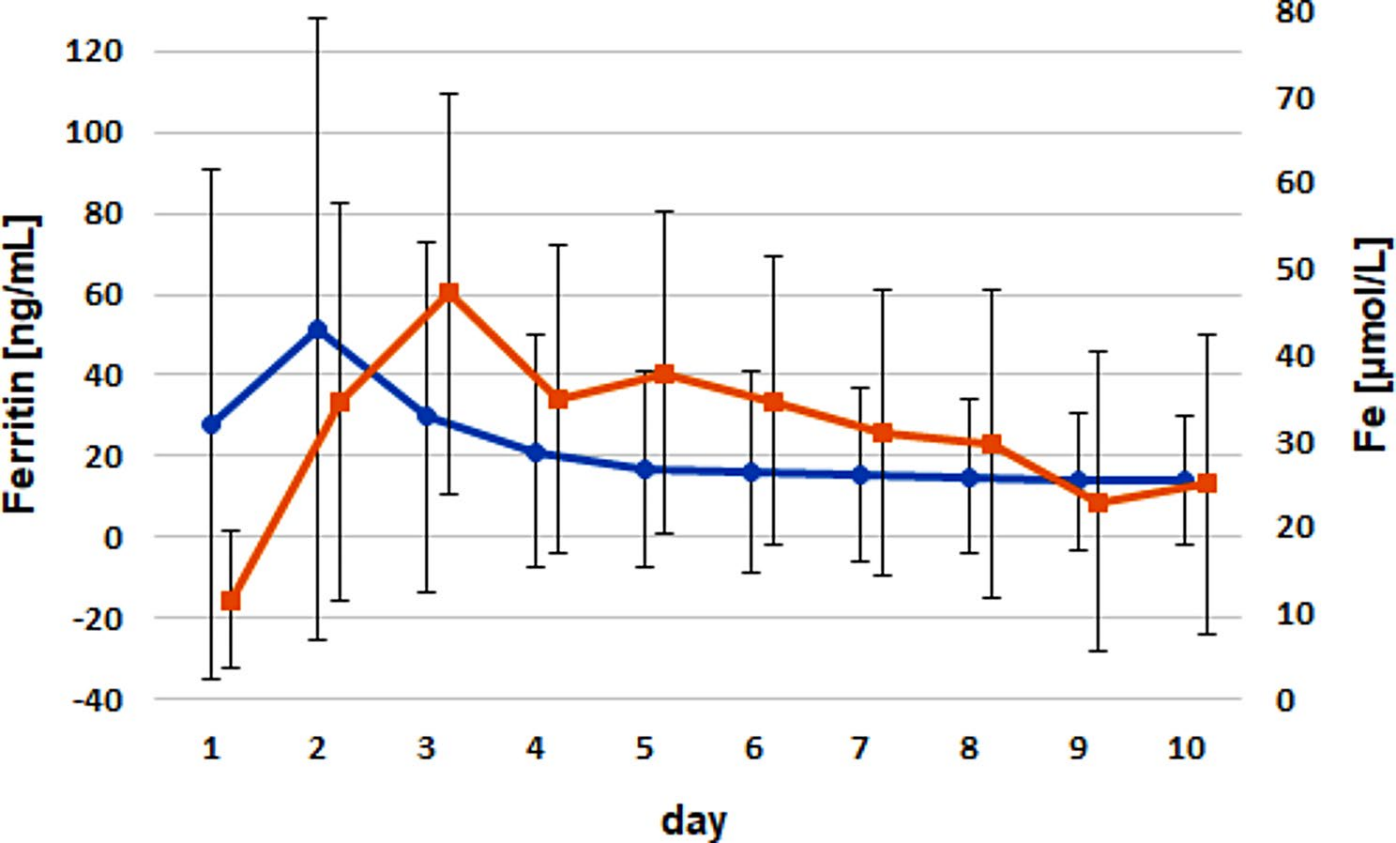
Comparison of the timely courses for ferritin (blue) versus serum iron (red) for both groups. Dots and triangles mark the arithmetic means; whiskers give the standard deviations. No significant correlations were identified

Time-dependent correlation analyses showed no significant correlation between ferritin and RBC, Fe (Fig. 2), Ht, or Hb in the examined calves during the first 10 days of life.

RBC counts declined mildly from birth until days 5 − 6 [treatment group: from 7.5 ± 0.96 T/L to 6.14 ± 0.84 T/L (day 5); control group: from 7.96 ± 1.4 T/L to 6.6 ± 1.2 T/L (day 6)] without dropping below the reference values. After reaching a minimum, the RBC count increased until the end of the examination period.

A similar curve progression was observed for the Ht and Hb levels. Ht reached its minimum at day 5 in the treatment group (day 1: 0.33 ± 0.05 l/L; day 5: 0.25 ± 0.04 l/L) and at day 7 (day 1: 0.34 ± 0.06 l/L; day 7: 0.27 ± 0.04 l/L) within the control group. The Hb curves showed a more pronounced decline after birth. Hb concentrations dropped steeply from day 1 to day 2 and showed a further but more even decline until days 5 or 6 in both groups (treatment group: day 1: 5.8 ± 0.95 mmol/L to day 2: 5.1 ± 0.98 mmol/L; control group: day 1: 6.2 ± 1.1 mmol/L to day 2: 5.5 ± 1.1 mmol/L).

**Fig. 3:**
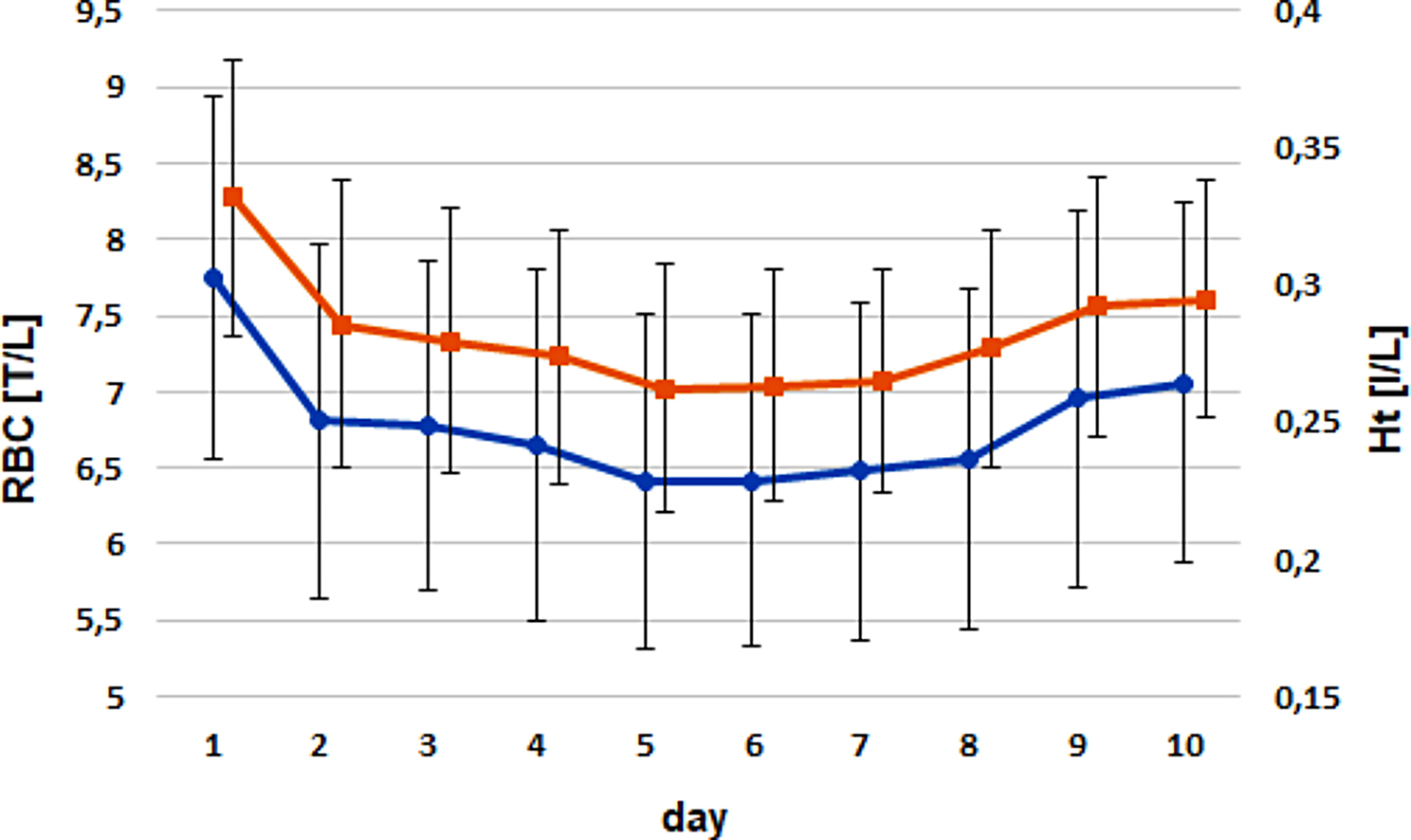
Comparison of the timely courses for RBC (blue) versus Ht (red) for both groups. Dots and triangles mark the arithmetic means; whiskers give the standard deviations

**Fig. 4:**
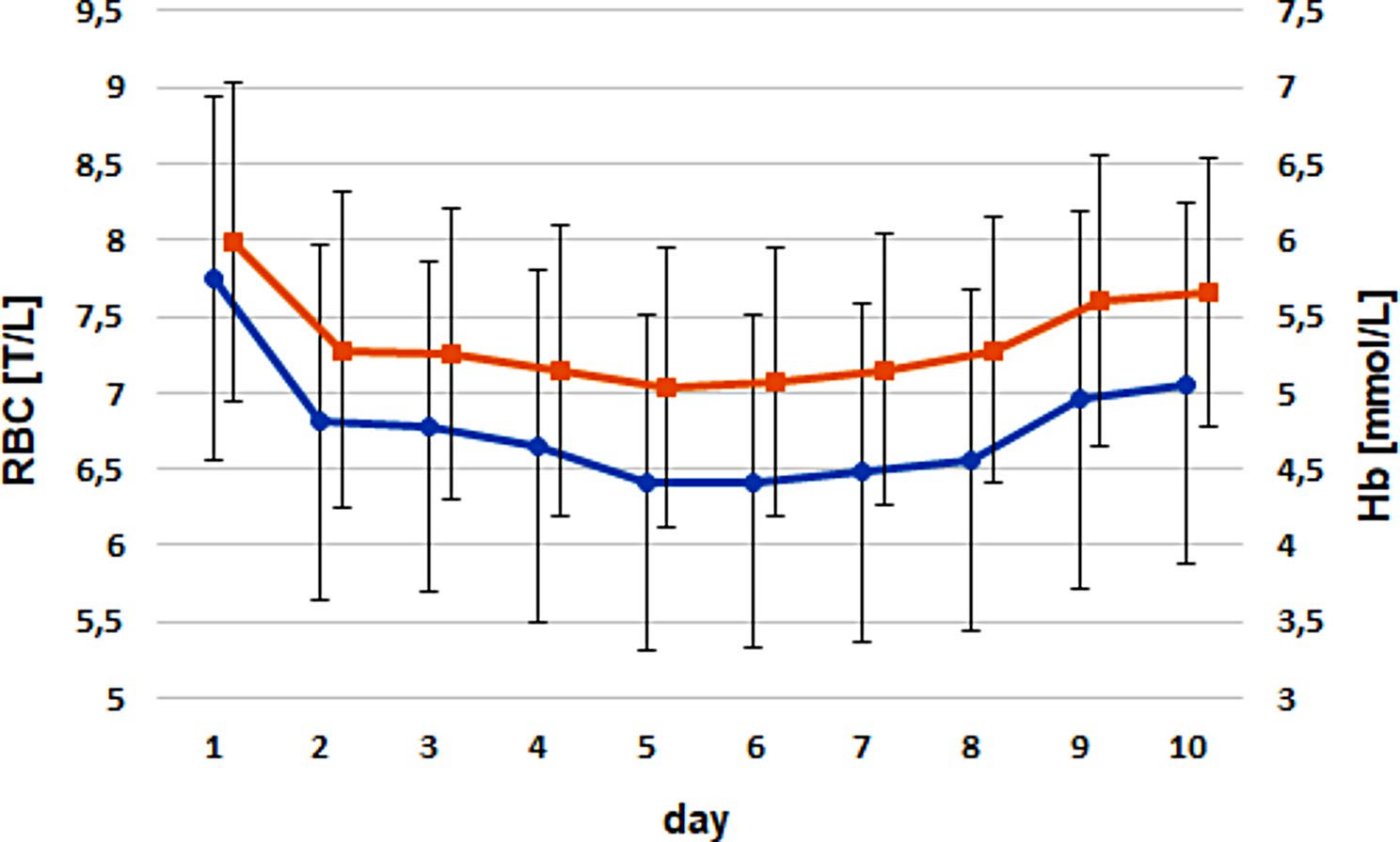
Comparison of the timely courses for RBC (blue) versus Hb (red) for both groups. Dots and triangles mark the arithmetic means; whiskers give the standard deviations

Strong positive correlations were observed for RBC versus Ht and Hb (ρ = 0.93 and ρ = 0.91; *P* < 0.0001; Figs. 3 and 4), as well as for the AUC values of these parameters. All correlations were independent of group or sex.

The AUC values for RBC, Ht, and Hb were influenced by treatment and health status but not by sex. Animals that received iron supplementation had significantly lower RBC count, Ht level, and Hb level than those of calves in the control group. Pairwise comparisons revealed that moderately diseased animals developed significantly higher RBC count, Ht level, and Hb level than severely diseased animals (Table 1). The erythrocyte indices were not influenced by any of the fixed effects tested.

**Table 1:** P-values for three-way ANOVA regarding RBC, Ht, and Hb

Source	RBC	Ht	Hb
Treatment	0.0342	0.0057	0.0170
Health status	0.0243	0.0097	0.0168
Sex	0.4204	0.1375	0.2454
Health status * treatment	0.7508	0.0884	0.1837
Health status * sex	0.1487	0.0664	0.1280
Treatment * sex	0.2891	0.3241	0.3919
Moderate vs. severe disease	0.0446	0.0109	0.0274

The pairwise comparison resulted in significant differences for moderate versus severe disease. ANOVA: analysis of variance; RBC: red blood cells; Ht: hematocrit; Hb: hemoglobin

Fe concentrations showed a marked increase from birth (treatment group: 9.8 ± 8.2 µmol/L; control group: 13.3 ± 7.5 µmol/L) until days 3 − 5 in both groups. Whereas in the treatment group, Fe concentrations peaked at day 3 (57.7 ± 23.4 µmol/L), the maximum iron concentration was reached on day 5 in the control group (37.5 ± 20.8 µmol/L).

Fe AUC values were significantly influenced by health status (*P* = 0.0227) but not by treatment (*P* = 0.2826) or sex (*P* = 0.7631). Healthy animals displayed significantly higher Fe concentrations than severely diseased animals (*P* = 0.0417).

**Fig. 5:**
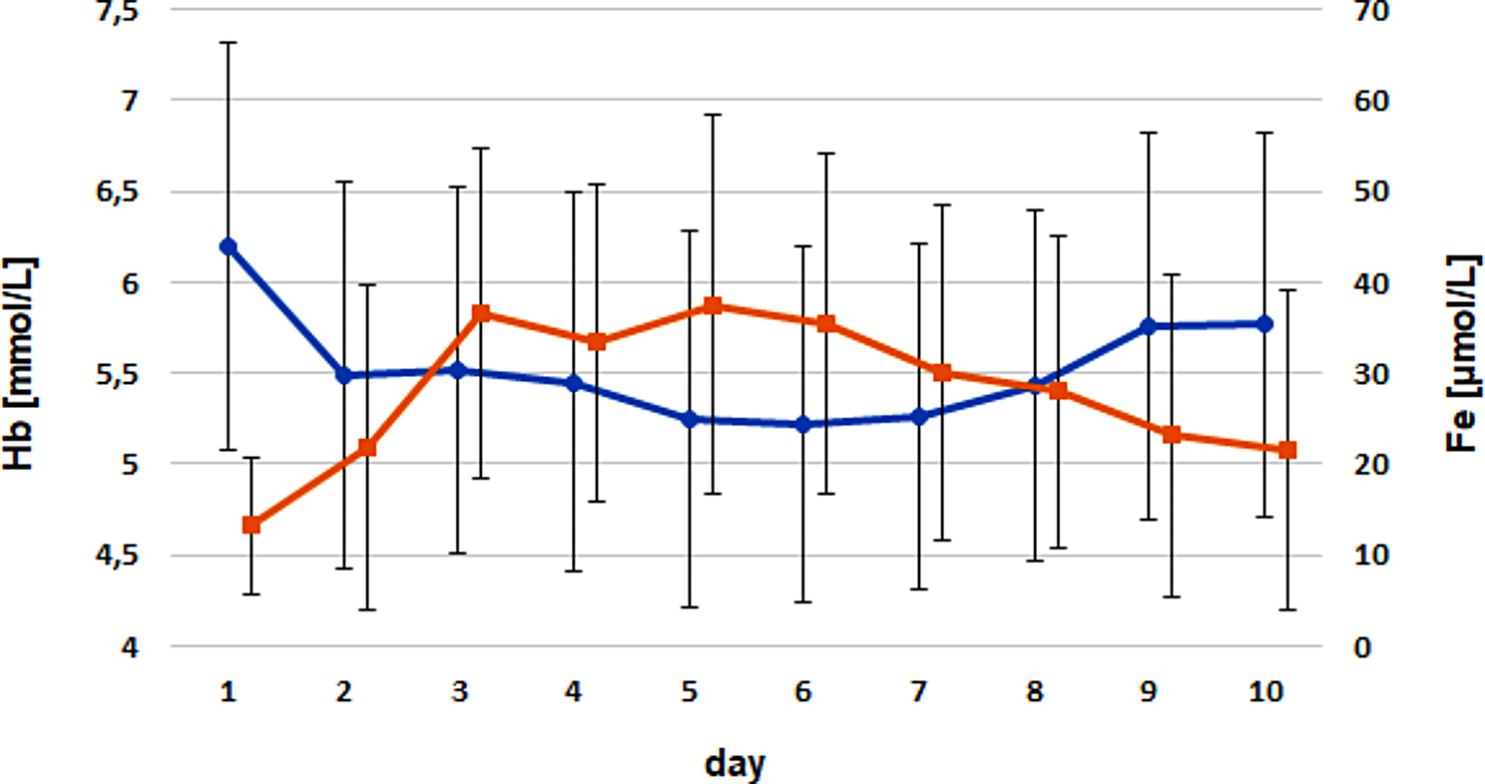
Timely course of Hb (blue) versus serum iron (red) within the control group (x ± s; weak negative correlation with *P* = 0.0087; Ρ=-0.57 at day 3)

Hb and iron concentrations showed a weak negative correlation at day 3 (*P* = 0.0034; ρ=-0.45), especially in the control calves (*P* = 0.0087; ρ=-0.57; Fig. 5). When grouping the concentrations per sex, a stronger negative correlation was observed for male animals (*P* = 0.0009; ρ=-0.68).

On day 3, correlation analysis showed a negative correlation between iron and RBC (*P* < 0.0001; ρ=-0.58). This correlation was independent of iron supplementation or sex. In female calves, a weak correlation between iron and RBC was observed on day 7 (*P* = 0.04; ρ = 0.47).

**Fig. 6:**
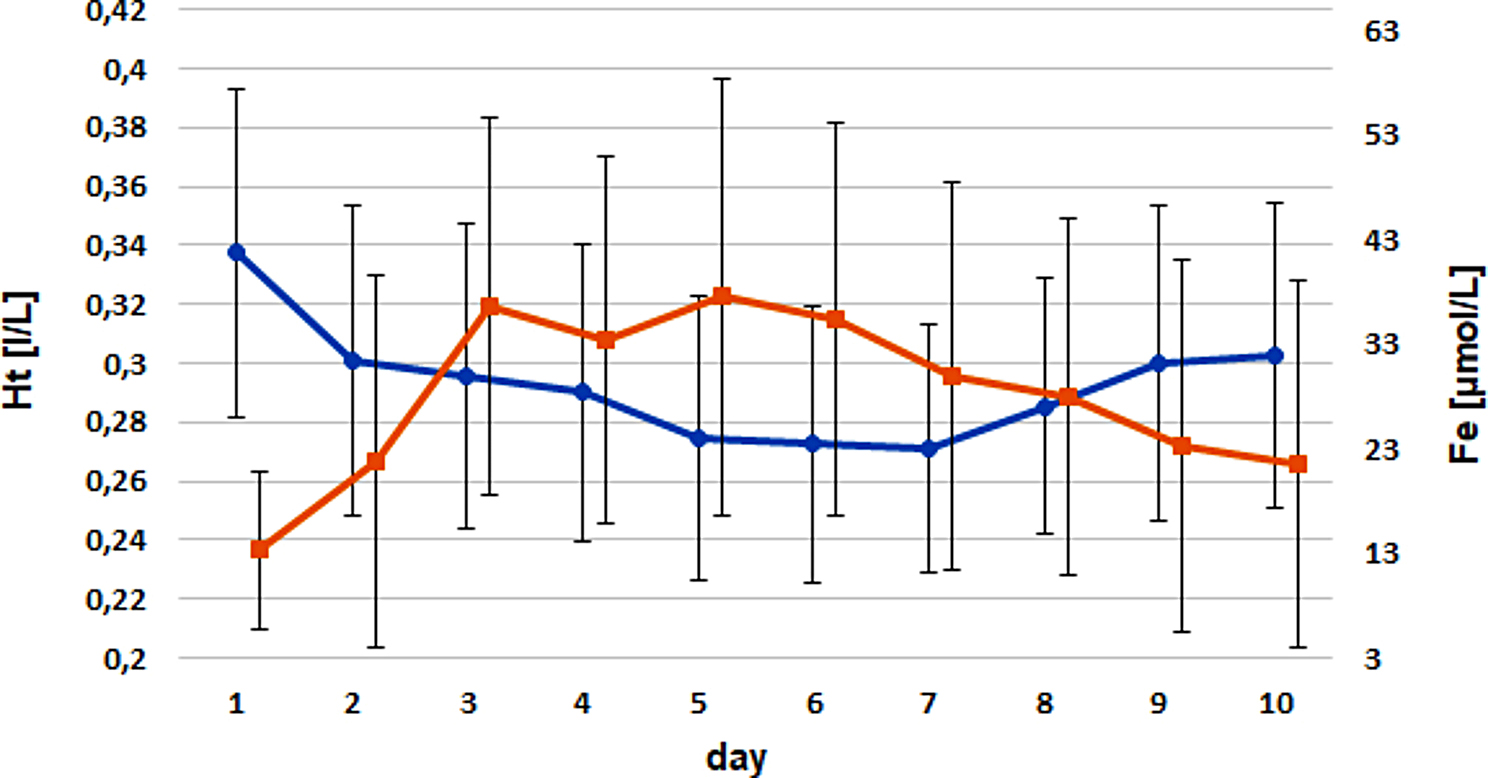
Timely course of Ht (blue) versus serum iron (red) within the control group (x ± s; weak negative correlation at day 3; *P* = 0.02; Ρ=-0.53)

Correlation analyses for Fe concentrations versus Ht levels revealed a weak, negative correlation at day 3 (*P* = 0.0032; ρ=-0.46), mainly in the control group (*P* = 0.02; ρ=-0.53; Fig. 6) and in male calves (*P* = 0.0016; ρ=-0.66). Fe concentrations increased from birth to day 3 and declined thereafter until day 10. Ht levels declined from birth until days 5–7 and increased thereafter.

Ferritin concentrations showed no relevant correlations with MCV, MCHC, or MCH.

### Health status

Based on clinical symptoms, eight calves were healthy throughout the examination period. Mild symptoms of diarrhea (*n* = 12) or thrombophlebitis (*n* = 3) were observed in 15 animals. Thrombophlebitis and diarrhea with a mildly elevated body temperature were classified as moderately impaired health status (*n* = 10). Calves with thrombophlebitis and watery diarrhea requiring treatment were considered severely ill (*n* = 7). Nine calves were treated with fluid therapy because of the development of diarrhea. None of the calves were administered antibiotics during the examination period. Nonsteroidal anti-inflammatory drugs (0.5 mg Meloxicam/kg; Melosolute^®^, CP-Pharma, Burgdorf, Germany or 40 mg Metamizol/kg; Metamizol WDT^®^, WDT, Garbsen, Germany) were administered to 12 of the 40 animals.

Signs of diarrhea were noted as early as day 2 (*n* = 6). Mild to severe diarrhea was noted in 13 calves from day 3 onwards and in 11 calves from day 6 onwards. Thickening of the jugular vein or elevation of body temperature was observed in two calves from day 3 onwards and in five calves from day 5 onwards.

### Study limitations

This study was designed to evaluate the effects of in-field iron supplementation under terms of a veterinary clinic and to examine serum ferritin as a potential marker for anemia in neonatal calves. The number of experimental calves used in this study required purchasing animals from different local farms. This heterogeneity concerning the origin of the animals induced a highly inhomogeneous situation regarding the immune status and fetal iron supplementation. The development of different diseases during the experimental procedure could be attributed to varying colostral quality, which may have influenced the results. Additionally, the calves with diarrhea were only examined parasitologically. Other infectious agents (i.e. *E. coli*, Rota- / Coronavirus) were considered negligible due to ongoing herd health control measures in the farms of origin. Coincidentally, the possibility of the presence of infectious agents negatively impacting the iron metabolism of some calves could not be ruled out. Explicitly, diagnostics to exclude the presence of iron-using bacteria would have been reasonable to eradicate the possibility of a negative impact on iron resorption, in addition to the inflammation of the intestinal wall during enteritis. Nonetheless, the presented results provide valuable insights into the follow-up developments of the examined parameters in very young calves.

## Discussion

Compared to Fe concentration, Ht, and Hb or RBC counts, individual serum ferritin concentration is regarded as a more suitable parameter to determine and evaluate iron-deficiency anemia because of its direct correlation with the iron storage of an organism [21, 26]. Fe concentrations underlie age-dependent fluctuations [21, 27–29], but hematological parameters and serum ferritin are thought to be influenced by a variety of factors, such as housing and nutrition [30], hydration, health status [31], or sex [20, 22, 32]. Therefore, it is unsurprising that the literature contains conflicting reference ranges and cutoff values for serum ferritin caused by differing analytical methods used to determine serum ferritin levels. Based on these data, a preliminary study used a cutoff value for serum ferritin of < 15 µg/L to monitor iron deficiency in calves of four different herds [33]. Although this was a preliminary study, the results showed that serum ferritin diagnosed anemia more reliably and earlier than Fe and Hb levels. Therefore, we elucidated and compared the temporal courses of the known parameters, Ht, RBC count, Hb concentration, erythrocyte indices, and Fe, with the serum ferritin concentrations in dairy calves within the first 10 days of life to evaluate their possible suitability as a more sensitive diagnostic tool for early indication of deficiencies. A possible influence of prophylactic iron supplementation and neonatal diseases, such as diarrhea and omphalitis, was evaluated.

However, the results of our study suggest that serum ferritin levels are not suitable as early diagnostic markers for neonatal calves. There were no significant correlations between ferritin levels and previously validated diagnostic markers of anemia in calves or adult cattle. Nevertheless, the interpretation of hematological parameters in neonates is complicated and sometimes misleading.

In neonatal calves, some physiological changes occur in hematological parameters, erythrocyte indices, and acid-base balance and should not be misinterpreted as pathological changes [7, 32, 34]. Within the first weeks of life, fetal Hb is replaced by adult Hb, Fe shows marked changes, and a reduction in MCV is observed without an underlying iron deficiency. Our observation of an elevation in serum ferritin within the first day of life aligns with previous studies in piglets and calves [21, 35] and might result from physiological alteration processes associated with neonatal erythrocytosis.

Interestingly, serum ferritin concentrations in our study were not influenced by the administration of iron via an intramuscular injection of iron dextran on the first day of life. In contrast, iron dextran injections (100 mg Fe) in nursing piglets resulted in a peak in serum ferritin at 1 week of age, which declined until 3 weeks of age [35]. In calves, iron dextran injections on day 3 and at the age of 2 weeks also led to an increase in serum ferritin concentrations within 1 week [21]. The observed differences between the aforementioned studies and our study might be due to a difference in serum ferritin levels at birth between the calves in the treatment and control groups in our study. However, concentration-dependent effects are plausible. In our study, the dosage consisted of 10 mg/kg iron dextran on day 1 of life, representing an often-used treatment pattern (injection of 5 mL of iron dextran solution per calf). A previously published study used comparable dosages, but not on the first day of life. Due to the different enzyme settings in neonates, treatment on the first day of life may not have the same results as treatment on day 3 [36]. Therefore, interference with the physiological increase in ferritin up to day 3 of life and possible pharmacodynamic differences between days 1 and 3 might have influenced the resorption of iron dextran, thus leading to a supplementation-independent elevation of serum ferritin. Therefore, a direct comparison between previous studies and the present study is critical. All the aforementioned studies reported marked inter-individual variances for serum ferritin, which might further complicate the interpretation of the presented results. However, the effects of the iron injection were observed in the treatment group. Fe levels were much higher in the treated animals than in the control animals, although the effect of iron supplementation was only visible until day 4.

A recent study examined the effects of different iron supplementation strategies (oral versus injection) on iron homeostasis in calves [37]. Although this study was conducted at a local research farm with its offspring and used a standardized amount of 1,000 mg of iron, our study investigated the effects of low-dose iron dextran supplementation (10 mg/kg i.m.) in an animal group that originated from different farms. These farms had below-average hygiene standards and were chosen to mimic the field conditions in our study, although the calves were transferred to the clinic. A low dose of iron dextran was used to examine the effect of the standard prophylactic treatment often used in the field to boost neonatal calves on the first day of their lives.

While Ht, Hb, and MCV are known to decline within the first weeks of life in young calves [7], neither their development nor that of other hematologic parameters have been evaluated within the first days of life, especially considering health status. RBC counts have been reported to be higher in young calves than in adult cattle, and a reduction in RBC count, Ht, Hb, and MCV confirms the diagnosis of anemia [38]. In our study, only four calves showed a reduction in RBC count, Ht, and Hb levels below their reference ranges. However, concerning the timely course, a mild reduction in these parameters was observed in all calves within the first 7 days of their lives. As previously shown in older calves [33], a significant positive correlation was observed.

In contrast to other studies [27, 34], we observed increasing concentrations of Fe independent of iron supplementation after birth. These differences may be the result of differences in the feeding habits of suckling calves. The animals in our study were nourished on a commercial milk substitute containing 150 mg/kg DM iron and forage, whereas Bostedt et al. [34] used calves fed whole milk only. Atyabi et al. [27] did not comment on the nutritional premises of their products. The possible oral iron intake of the calves in our study was higher than that of calves fed whole milk only, thus resulting in an elevation of Fe levels independent of supplementation within the first days of life. This is consistent with the results of Golbeck et al. [37]. The calves used in the present study also showed an increase in estimated transferrin saturation at the end of the first week of life. We concluded that this increase resulted from feeding with a conventional milk substitute.

While our results suggest a negative effect of iron supplementation on the development of Ht, Hb, and RBC, we must consider a possible bias due to the adverse distribution of calves between the treatment and control groups. Although the calves were randomly allocated to the groups, the calves in the treatment group had lower average values of nearly all parameters on day 1 compared to those in the control group. Timely developments in Ht, Hb, and RBC counts did not differ between the groups. Consequently, the statistically significant influence of iron supplementation on these parameters was not apparent clinically. Therefore, in line with other studies, this warrants a critical evaluation of the necessity for iron supplementation [37, 39, 40].

In contrast, the effects of diseases were not biased because equal numbers of animals in both groups developed signs of disease during the study protocol. As observed in previous studies [9, 12, 13], severely diseased animals had reduced Fe concentrations compared to healthy animals. This physiological metabolic pathway may be seen as a counter-regulation of the body against pathogens [8]. The effects of diseases on the hematological parameters, Ht, Hb, and RBC, were consistent. Animals with moderate signs of disease showed higher levels of Ht, Hb, and RBC. However, the necessity of fluid therapy in severely diseased animals may explain these effects as dilution effects [41].

Another important aspect that warrants mention is that iron absorption is impaired in intestinal inflammation [42] resulting in hypoferremia at an early stage of infection [43]. Siderophilic bacteria, such as *Salmonella*, *Clostridia*, and some pathogenic *E.coli*, further influence iron absorption, because these enteric pathogens compete for Fe^2+^, the absorbable oxidation state of iron [44]. Modulatory innate defense mechanism and iron deprivation via bacterial infection might have contributed to the reduced serum iron concentrations in the present study. Hence, the results of the present study must be considered limited, due to the fact that no microbiological examinations were performed that could have validated or eradicated the presence of the aforementioned siderophilic enteropathogenic bacteria.

In general, Fe and serum ferritin are reportedly influenced by inflammation [45], resulting in reduced suitability as iron deficiency indicators. However, serum ferritin is still considered the most reliable biomarker of total body iron stores in cases of minor inflammation [45]. In the present study, serum ferritin concentrations showed no dependence on the presence of disease, although various inflammatory diseases, such as diarrhea, thrombophlebitis, and omphalitis, occurred during the course of the study protocol. As serum ferritin itself is a positive, acute-phase protein [46], it was assumed that the inflammatory status in the animals of the present study was rather mild, and therefore, no effects of the disease could be demonstrated. However, correlation analyses targeting regression corrections were not performed. In preschool children, this approach resulted in a 25% higher estimated prevalence of iron deficiency based on serum ferritin levels [46]. Unfortunately, no data interpreting iron indicators using inflammatory biomarkers in neonatal calves are available; this should be assessed in future studies.

## Conclusion

The results of this study revealed that serum ferritin does not appear to be a useful parameter for evaluating iron storage in neonatal calves. Due to its stable course from day 4 onwards, the diagnostic value of serum ferritin in older calves or adult cattle with anemia must be further elucidated. Iron dextran injections elevate Fe concentrations, but there seems to be no immediate effect on iron storage, as evaluated by serum ferritin concentrations. The often-used standard supplementation of iron to neonatal calves may, therefore, be critically scrutinized. Sex-associated differences concerning the correlations between the examined parameters of this study were negligible, and any influence of sex indicated a slightly higher risk for male calves to suffer from alterations in iron metabolism. The statistically significant effects of impaired health status on Fe concentrations were apparent. The influence of iron supplementation or impaired health status on hematological parameters was present but rejected due to bias or therapeutic influence.

## Methods

### Animals

This study was approved by the Giessen Regional Council (V 54 − 19 c 20 15 h 01 GI 18/14; Nr. G 79/2019) and adhered to ARRIVE 2.0 standards. The study was conducted between February 2020 and September 2021 and included 20 male and 20 female German Holstein neonatal calves. Thirty-eight calves were purchased from dairy farms in Hessen, Germany, located at a radius < 15 km from the Justus-Liebig-University in Giessen, and two calves were from spontaneous deliveries at the Clinic of Obstetrics, Gynecology and Andrology for Small and Large Animals of the Justus-Liebig-University.

### Housing and feeding

Immediately after birth, the calves were separated from their dams and fed 2–3 l of colostrum from the dam, depending on their body weight. Calves were housed individually in boxes bedded with straw from birth to 10 days of age and moved into group pens thereafter. After the second colostrum meal, the calves were fed 2 l of a commercial milk substitute three times daily (CombiMilk Start, Agravis Raiffeisen AG; Fe(II): 150 mg/kg DM). Roughage (hay), concentrated feed (Kälberkraft; Agravis Raiffeisen AG), and free access to water were provided from the second day of life.

### Experimental procedures

Shortly after birth, the calves underwent a complete clinical examination to confirm their suitability for the study, including assessments of clinical health (e.g., appearance, heart rate, respiration, and suckling reflex), checks for congenital malformations or a short-ripped navel, and their first blood collection, via jugular venipuncture (18 G cannula). Male and female calves were randomly assigned to two groups. Group A served as the control and did not receive any substitutes with trace elements, minerals, or vitamins. Calves in group B were administered 10 mg/kg Fe^3+^ as an iron (III)-hydroxide-dextran complex (Belfer^®^ iron dextran, bela-pharm GmbH & Co. KG, Vechta, Germany) intramuscularly on the first day of life.

Further blood sampling was performed through a jugular vein access (PUR Infusionskatheter^®^, 16 G, softtip, length 15 cm; Walter Veterinär-Instrumente e.K., Baruth, Germany) placed on the second day of life. The standardized time for sampling was in the morning after the first milk meal over the first 10 days of life and was accompanied by a clinical examination (e.g., behavior, rectal temperature, fecal consistency, palpation of the navel, and joints). Access to the jugular vein was examined twice daily for signs of inflammation. Blood samples were collected in serum-separating tubes containing EDTA (ethylenediaminetetraacetic acid) and lithium heparin (Kabe; Nürmbrecht, Germany). Serum was separated from whole blood via centrifugation (20 min, 1,000 × G) and stored at -80 °C until use.

### Blood parameters

RBC (reference range, 5–10 T/L) and Hb (reference range, 5.6–8.7 mmol/L) were measured using the cell counter IDEXX ProCyte Dx (IDEXX laboratories, Kornwestheim, Germany). Additionally, Ht (reference range, 0.28–0.38 l/L) was measured after centrifugation (Haematokrit 210; Hettich, Kirchlengern, Germany). MCV (reference range, 27–36.1 fL), MCH (reference range, 0.66–0.84 fmol), and MCHC (reference range, 20.2–24.18 mmol/L) were calculated after the determination of Hb, Ht, and RBC. Total protein (reference range, 40–60 g/L) was measured daily. On day 2 of life, the cutoff value for remaining in the study was 54 g/L, indicating sufficient colostrum intake.

WBC counts were determined but are not further addressed here to retain emphasis on the indicators of anemia. Further details may be available upon request from the authors.

Fe level (reference range, 14.5–25 mmol/L, Test kit LT-SI 0100; Labor und Technik Lehmann GmbH) was measured photometrically at the specific wavelength recommended by the manufacturer. Ferritin levels were determined using bovine ELISA (enzyme-linked immunosorbent assay) plates (Cloud-Clone Corp., Texas, United States). All samples were measured in duplicate to determine ferritin concentrations.

### Health status

All animals were clinically healthy on day 1 of the study period. They were clinically examined once daily to monitor their demeanor, heart and respiratory rates, inner body temperature, signs of diarrhea or pneumonia, and possible inflammatory reactions with respect to the permanent jugular vein catheter or the navel. According to the clinical symptoms and necessity for treatment, the calves were categorized based on the occurrence of diseases as healthy or showing mild, moderate, or severe signs of disease. Calves were classified as diseased if their vital parameters were altered and signs of diarrhea, omphalitis, or thrombophlebitis were present. None of the calves showed signs of pneumonia. Diseases were scored based on the number of days with signs of disease as healthy (0–2 days), mild (2–5 days), moderate (5–7 days), or severe (7–10 days).

In cases of diarrhea, parasitological examinations were performed using fecal smears stained with carbol fuchsin to test for cryptosporidiosis. Further parasitological diagnostics, microbiology, or virological examinations were not performed due to ongoing herd health management measures in the farms of origin. The risk of the presence of pathogenic bacteria or virological agents was considered limited.

### Statistics and data analyses

The Department for Biomathematics and Data Processing of the University of Giessen performed the power analysis and the statistical data analyses. The power analysis was performed using BiAS Version 9 (Epsilon publishing, Hochheim, Darmstadt, Germany). All statistical analyses were performed using SAS^®^ 9.4 (SAS Institute Inc., 2013. Base SAS^®^ 9.4 Procedures Guide: Statistical Procedures, 2nd edition ed., Statistical Analysis System Institute Inc., Cary, NC, USA). The AUCs for ferritin, Ht, RBC, Hb, MCV, MCH, MCHC, and Fe were determined. All AUC values were logarithmized to achieve a normal distribution. Values were compared using a three-way analysis of variance to evaluate the possible influence of iron treatment, health status, and sex on these values. The Bonferroni adjustment was used for multiple comparisons. The time-dependent developments of all examined parameters within the examination period (10 days) were displayed and compared graphically after grouping animals according to treatment or sex.

Spearman correlation analyses were performed to reveal possible correlations between the parameters. Significance was set at values of P < 0.05.

## Data Availability

The data that support the findings of this study are not openly available due to reasons of sensitivity and are available from the corresponding author upon reasonable request.

## Abbreviations

Ht: hematocrit
RBC: Red blood cells
Hb: Hemoglobin
MCV: Mean corpuscular volume
MCH: Mean corpuscular hemoglobin
MCHC: Mean corpuscular hemoglobin concentration
Fe: Serum iron
DM: Dry matter
AUC: Area under the curve
ELISA: Enzyme-linked immunosorbent assay
ANOVA: analysis of variance
